# Methods for the Discovery and Identification of Small Molecules Targeting Oxidative Stress-Related Protein–Protein Interactions: An Update

**DOI:** 10.3390/antiox11040619

**Published:** 2022-03-23

**Authors:** Xuexuan Wu, Qiuyue Zhang, Yuqi Guo, Hengheng Zhang, Xiaoke Guo, Qidong You, Lei Wang

**Affiliations:** 1 State Key Laboratory of Natural Medicines and Jiangsu Key Laboratory of Drug Design and Optimization, China Pharmaceutical University, Nanjing 210009, China; 3219010067@stu.cpu.edu.cn (X.W.); 3119010019@stu.cpu.edu.cn (Q.Z.); 2020202340@stu.cpu.edu.cn (Y.G.); 3320011089@stu.cpu.edu.cn (H.Z.); 2 Department of Medicinal Chemistry, School of Pharmacy, China Pharmaceutical University, Nanjing 210009, China

**Keywords:** Nrf2, oxidative stress response, protein-protein interaction

## Abstract

The oxidative stress response pathway is one of the hotspots of current pharmaceutical research. Many proteins involved in these pathways work through protein–protein interactions (PPIs). Hence, targeting PPI to develop drugs for an oxidative stress response is a promising strategy. In recent years, small molecules targeting protein–protein interactions (PPIs), which provide efficient methods for drug discovery, are being investigated by an increasing number of studies. However, unlike the enzyme–ligand binding mode, PPIs usually exhibit large and dynamic binding interfaces, which raise additional challenges for the discovery and optimization of small molecules and for the biochemical techniques used to screen compounds and study structure–activity relationships (SARs). Currently, multiple types of PPIs have been clustered into different classes, which make it difficult to design stationary methods for small molecules. Deficient experimental methods are plaguing medicinal chemists and are becoming a major challenge in the discovery of PPI inhibitors. In this review, we present current methods that are specifically used in the discovery and identification of small molecules that target oxidative stress-related PPIs, including proximity-based, affinity-based, competition-based, structure-guided, and function-based methods. Our aim is to introduce feasible methods and their characteristics that are implemented in the discovery of small molecules for different types of PPIs. For each of these methods, we highlight successful examples of PPI inhibitors associated with oxidative stress to illustrate the strategies and provide insights for further design.

## 1. Introduction

Oxidative stress (OS) is a state of imbalance between the production of reactive oxygen species (free radicals) and antioxidant defenses, that can become a main trigger of inflammation, aging, and chronic diseases including cancer [1,2]. Free radicals are generated in many biochemical processes or in some instances as activated neutrophils. In addition, the organism is exposed in a free radical-rich environment, such as electromagnetic radiation, ozone, and nitrogen dioxide. The complex antioxidant defense mechanism protects the body from free radical attack. Once antioxidant defenses are disturbed or deficient, tissue injuries will occur [3,4]. Oxidative stress will induce changes in multiple cytokines and the antioxidant defense mechanism will mobilize diverse signal factors to counteract oxidative stress. For example, oxidative stress can activate the MAPK signaling pathway that induces activation of ERK, JNK, and p38 kinase, which can respond to oxidative stress by promoting the production of reactive oxygen species (ROS) [5]. Oxidative stress induces HSP90 upregulation that becomes a promising target for autoimmune reactions [6]. Nuclear factor erythroid 2-related factor 2 (NRF2) is a transcription factor that regulates the expression of antioxidant and detoxification genes. In addition, interleukin-2 (IL2), programmed cell death-1 (PD-1), p53, and B-cell lymphoma 9 (BCL9) are also closely related to oxidative stress [7,8,9,10,11,12]. Hence, these proteins are promising targets for the development of antioxidants [13,14].

**Figure 1:**
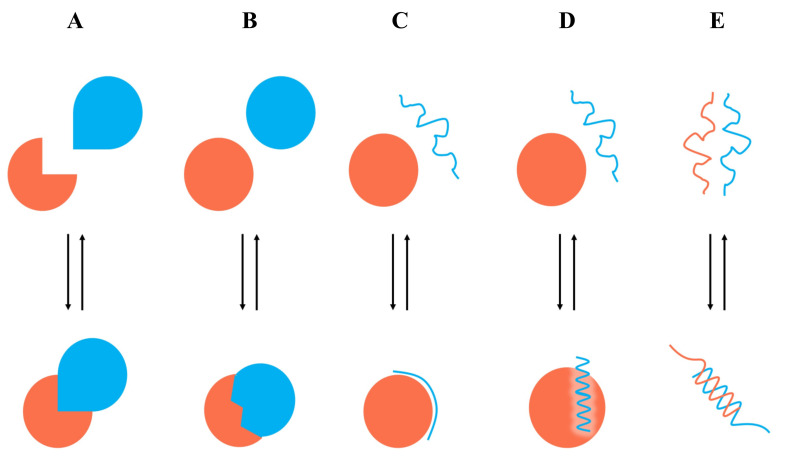
Classification of protein–protein interactions. (**A**) Type I, two proteins interact through preformed surfaces. (**B**) Type II, two globular proteins interact through an induced binding surface. (**C**) Type III, a rigid globular protein interacts with a peptide. (**D**) Type IV, a flexible globular protein interacts with a peptide. (**E**) Type V, interaction between two peptide chains.

Proteins work by forming different complexes, which involve multiple protein–protein interactions (PPIs) and play important roles in the regulation of biological systems [15]. However, the abnormal regulation of PPIs is closely related to diseases. The rational design of small molecules to regulate PPIs is emerging as one of the most important ways to study protein functions and mechanisms. However, the discovery of small molecules that directly disrupt the rigid interactions between two proteins is extremely challenging and even considered “undruggable” in certain cases [16]. For the past decade, the “hot-spots” theory guided scientific investigations into the majority of binding forces on PPI interfaces. In particular, small molecules were designed to bind to “hot-spots” and directly disrupt the interaction between two proteins [17]. Thus, it was feasible to optimize small molecules based on the characteristics of well-defined binding pockets on “hot-spots”. Many successful drugs and drug candidates have been developed based on this theory, including ABT263, RG7112, AMG232, MI-77301, SAR1118, etc. [18]. Although fruitful results have shown promising prospects, there are more cases of different types of PPIs (lack of available small molecules) that remain unclear, owing to the limited structural biology information as well as biological screening techniques [19]. In addition, early PPI screening methods are prone to be artifacts and tend to choose hydrophobic compounds with non-drug-like mechanisms of action, such as aggregation-based inhibition or protein denaturation [20]. Generally, structural information of PPIs, including X-ray and NMR results, is extremely important for drug discovery. PPIs typically exhibit dynamic features and can be classified into certain classes. In particular, interactions between two proteins can be described by five models (Figure 1): type I, pairs of globular proteins that interact through a discontinuous epitope with no substantial structural changes on binding; type II, proteins with preformed globular structures that adapt upon interaction to form a complex with a novel conformation; types III and IV, PPIs involving globular proteins (rigid globulin or flexible globulin) that interact with a single peptide chain; and type V, with PPIs between two peptide chains [21]. There is an urgent need to develop more rational techniques for the discovery of small molecules that target PPIs.

Currently, many methods, ranging from protein-based molecular methods to cell-based high-content screening strategies, have been developed to identify compounds that interfere with PPIs of interest [22]. Ideally, regardless of the type of PPI, high-throughput screening (HTS) methods are promising. Herein, we outlined the current methods that are used to identify small molecules that target PPIs, based on five classes (proximity-based, affinity-based, competition-based, structure-guided, and function-based methods). In this review, we not only focus on the principles of each method, but also provide evidence of the available PPI types that can be targeted. Moreover, we discuss the merits and limitations of each strategy, as well as defined cases in which each method has been applied. Thus, a summary of the assays and techniques that could be applied to characterize small molecules targeting oxidative stress-related PPIs for future antioxidant drugs design is outlined.

## 2. Proximity-Based Methods

**Figure 2:**
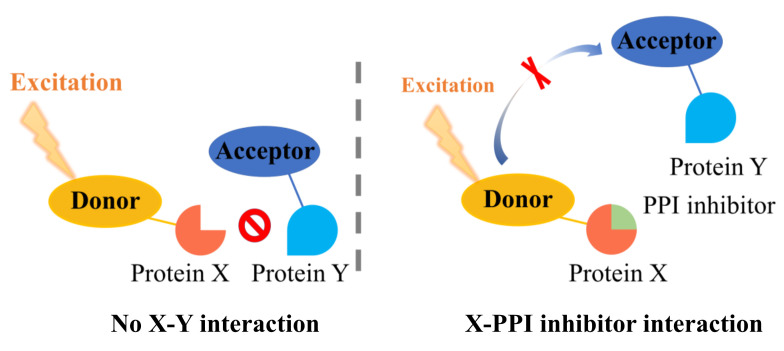
Principles of the proximity-based method. X and Y protein are labeled with donor and receptor moieties, respectively. Once the X–Y interaction is formed, the receptor will accept a signal from the donor and produce a detectable signal. A PPI inhibitor will block the interaction between X and Y protein, thus preventing signal generation.

Based on the nature of PPI binding modes, the rational design of proximity-based methods is becoming a promising strategy for discovering PPI inhibitors. All reported assays have been developed based on the interactions between two proteins, and the principle of proximity-based methods is shown in Figure 2. Generally, X and Y proteins are labelled with donor and receptor moieties, respectively. Once the X-Y interaction is formed, the receptor accepts a signal from the donor and produces a detectable signal. According to different donor and acceptor excitation and emission mechanisms, these assays can be divided into amplified luminescent proximity homogeneous assay (alpha) technology and fluorescence resonance energy transfer (FRET) technology.

### 2.1. Alpha Technology

**Figure 3:**
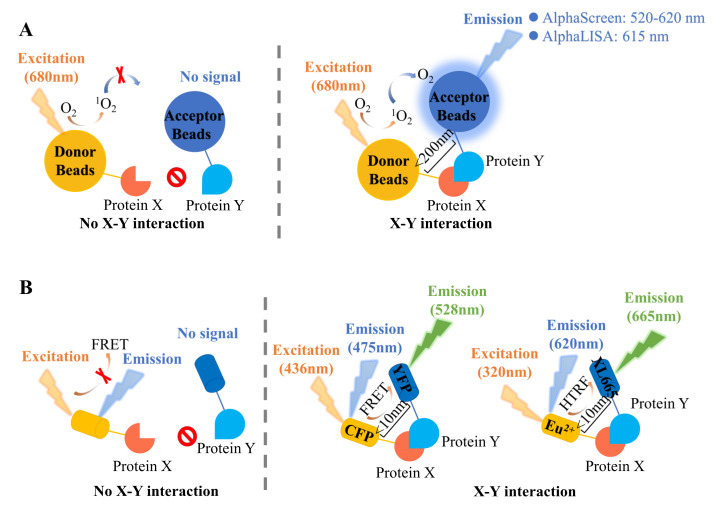
Illustration of representative proximity–based methods. (**A**) Principles of alpha technology. Proteins X and Y are immobilized on donor and acceptor beads, respectively. The photosensitizers in the donor bead convert oxygen (O_2_) into singlet oxygen (^1^O_2_) when it is excited by light at 680 nm. If there is no X–Y interaction (distance between the donor bead and the acceptor bead is greater than 200 nm), the singlet oxygen will fall back to ground state, without any light signal generation. If an X–Y interaction occurs, singlet oxygen initiates a cascade of energy transfer steps in the acceptor bead, resulting in the generation of light emission at 520–620 nm (in AlphaScreen) or 615 nm (in AlphaLisa). (**B**) Principles of Förster resonance energy transfer (FRET). Proteins X and Y are fused with donor and acceptor fluorophores, respectively. In the absence of an X–Y interaction (distance between the donor and the acceptor is greater than 10 nm), donor fluorophore excitation results in light emission from the donor alone. When an X–Y interaction occurs, the donor and acceptor fluorophores are brought into proximity, resulting in energy transfer from the donor to acceptor and acceptor emission. In FRET, the donor and acceptor fluorophores are CFP and YFP, respectively, and the detectable emission light is at 528 nm. In TR-FRET and HTRF, the donor fluorophores are terbium and europium cryptates, respectively. Both acceptors use an XL665 donor fluorophore to generate emission light at 665 nm.

The amplified luminescent proximity homogeneous assay (alpha) system is a bead-based technology that has been developed from the luminescent oxygen channeling immunoassay (LOCI) diagnostic assay methodology, and used to study interactions between two proteins and for screening purposes [23]. As shown in Figure 3A, the key components of this technology are chemical-containing donor and acceptor beads that can bind to proteins of interest [24]. The donor bead contains a photosensitizer, phthalocyanine, which can convert oxygen (O_2_) into singlet oxygen (^1^O_2_) upon excitation by light at 680 nm. Each donor bead can produce about 60,000 singlet oxygen molecules, which can diffuse to a distance of 200 nm in solution, within a half-life of 4 μs [25]. Three chemical dyes are coated in an acceptor bead that performs a tandem reaction that is triggered by singlet oxygen [24]. Based on the different acceptor bead compositions, alpha technology is further divided into two types, namely AlphaScreen and AlphaLISA. AlphaScreen acceptor beads are embedded with thioxene, anthracene, and rubrene [26]. Within 200 nm of the donor bead, singlet oxygen firstly reacts with thioxene to generate light [27]. Then, this light energy is transferred to anthracene and rubrene. Finally, a broad detectable light signal, from 520 to 620 nm, is emitted by rubrene [28,29]. In the absence of acceptor beads, the singlet oxygen will fall back to ground state, without any light signal generation [24,28]. In AlphaLISA acceptor beads, anthracene and rubrene are replaced by europium (Eu) chelate, which, upon excitation, emits an intense light with a much narrower wavelength bandwidth than AlphaScreen centered around 615 nm. This emission range can mitigate interference by some compounds that absorb light between 500–600 nm [24]. Based on the above principles, alpha technology has been widely used in the discovery of PPI inhibitors and has become an efficient HTS method.

Currently, alpha technology is commonly used in biochemical research and drug discovery, owing to its following unique features. As shown in Table 1: (1) Simple operation: this assay is performed in a solution and does not require any washing steps. (2) Broad energy transfer distance: Compared with TR-FRET, whose maximum transfer distance is ~10 nm, the distance between donor and acceptor beads is 200 nm or more, which enables the study of larger protein–protein complexes. (3) High sensitivity: Under excitation at 680 nm, each donor microbead can release 60,000 singlet oxygen/s, generating very high signal amplification. In addition, the wavelength of the read signal is lower than the excitation wavelength, where most of the background signal is generated. Thus, this technology can be used for low-(pM) to high-affinity (mM) interactions. (4) High throughput: It can be used for HTS in 96-, 384-, or 1536-well assay formats. (5) Wide applicability: It can be used in biochemical and cell-based assays due to its satisfactory compatibility with complex matrices such as cell lysates, serum, and plasma. Nevertheless, certain limitations remain that deserve further attention. First, the donor beads are light sensitive. Direct sunlight or intense artificial light can activate donor beads and induce light emission from acceptor beads. Second, the signal is temperature-dependent. The interaction between antibodies and analytes is influenced by incubation temperature, and the generation rate and diffusion of singlet oxygen are affected by the temperature of the plate reader. Third, each alpha bead has a maximum binding force and “hook effect”, which is an intrinsic drawback of the sandwich method (such as ELISA experiments). Therefore, the alpha technology cannot be applied for enzyme kinetic analysis [30]. 

### 2.2. Fluorescence Resonance Energy Transfer (FRET)

Fluorescence resonance energy transfer (FRET) is commonly used in PPI studies [31]. It was first proposed by Theodor Förster in 1946 and has been applied to biological and diagnostic systems since the 1970s [32]. FRET involves nonradiative (dipole–dipole) energy transfer between two fluorophores, called (energy) donor and (energy) acceptor, respectively [33]. As shown in Figure 3B, when the donor is excited by an external energy source, a specific wavelength of light is emitted. If the donor is within a sufficient distance from the acceptor (usually 1~10 nm), the emission energy of the donor excites the acceptor, and new emission light from the acceptor is generated [34]. Upon energy transfer, the fluorescence signal of the donor decreases, while the fluorescence signal of the acceptor increases. In general, fluorescent proteins or small organic dyes are selected as fluorophore pairs, such as Alexa Fluor 488-Alexa Fluor 546, Alexa Fluor 568-Alexa Fluor 647, cyan fluorescent protein (CFP)-yellow fluorescent protein (YFP), and mVenus-mStrawberry [35,36,37]. Based on FRET, time-resolved FRET (TR-FRET) has been developed with long-life donor lanthanide chelate fluorophores (such as europium and terbium chelate) that permit a time delay between excitation and measurement, to avoid interference from short-lived background fluorescence [38,39]. With further improvements, homogeneous time-resolved fluorescence (HTRF) has been established with higher detection sensitivity and stability and has become the most widely used proximity-based method [38]. Europium and terbium cryptate donors are used in HTRF for their longer emission lifetime (1–2 ms) than conventional fluorophores (1~50 ns) [40]. This greatly reduces background fluorescence interference and avoids false positives during screening. In addition, in the lanthanide cryptate, europium or terbium is stably embedded within holes. Because the energy transformation efficiency is distance-dependent (within the 1–10 nm range), HTRF is highly sensitive for investigating PPIs both in vitro and in vivo.

Bioluminescence resonance energy transfer (BRET) also belongs to the protein interaction research method of energy transfer. The main difference between it and FRET is that FRET involves energy transfer between two fluorophores, one of which requires an external light source. While BRET occurs after the substrate is oxidized and does not require external excitation. Therefore, BRET has many advantages over FRET, such as no excitation light, lower background, and avoids photobleaching and autofluorescence. In BRET technology, protein A is fused to luciferase as an energy donor, and protein B is fused to a tagged protein with a fluorescent group as an energy acceptor. If there is an interaction between protein A and B, after adding the luciferase substrate, the luciferase luminescence can excite the fluorophore of the adjacent protein B to emit light [41].

This type of technology, particularly TR-FRET and HTRF, has been widely used in recent scientific research. The advantages are summarized as follows: (1) Simple operation without washing. (2) Stability: The solution system can be monitored repeatedly for at least 48 h. (3) Low interferences: The low background signal significantly decreased the probability of false positives and negatives. (4) Wide range of applications. (5) Simple miniaturization for high-throughput screening. The main limitation of this technology is its low dynamic range, which hinders its application to larger proteins (Table 1).

## 3. Affinity-Based Methods

Affinity-based methods are widely used in drug discovery processes to provide detailed binding affinities for characterizing the activity of a compound. For small molecules that target PPIs, it is important to ensure that their binding affinity is well defined. Such results offer important evidence for avoiding false-positive results. In addition, the correlation between the binding affinity and IC_50_ values also provides significant clues for the optimization of small molecules. Currently, many affinity-based methods with different principles are being utilized (Table 2). Each of these has advantages and disadvantages. Notably, affinity-based methods can be used for all of the PPI types.

### 3.1. Thermal Shift Assay

**Figure 4:**
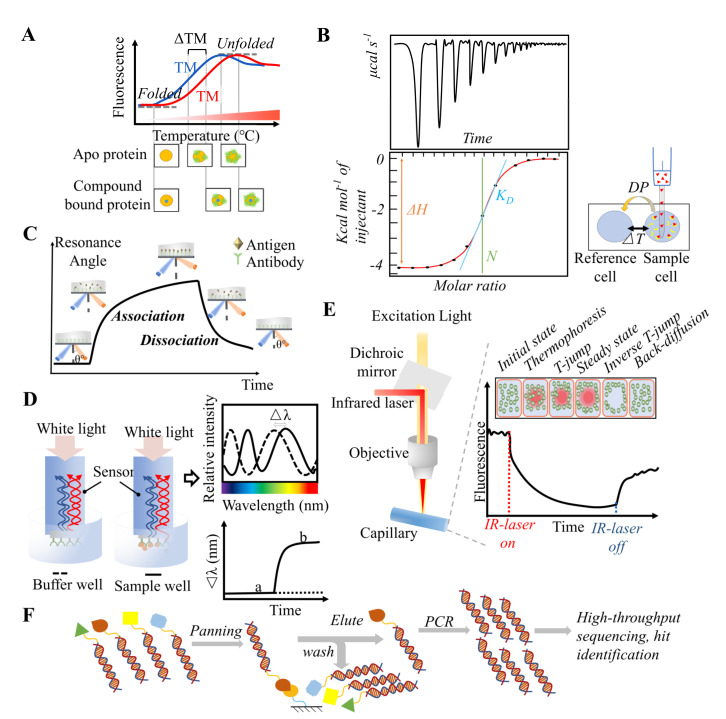
**Illustration of affinity**−**based methods.** (**A**) Thermal shift assay (TSA). (**B**) Isothermal Titration Calorimetry (ITC). ITC instruments are composed of a sample cell and a reference cell, both of which are coated by an adiabatic jacket. The temperature difference between the two cells is detected by a connected sensitive thermocouple circuit during the titration process. (**C**) Surface plasmon resonance (SPR). The protein is first immobilized onto the sensor chip by covalent coupling or interaction with specific fusion tag-containing antibodies. Then, the ligand solution flows across the chip surface in the microfluidic channel and continuously binds to the protein. This results in a change in the refractive index of the metal surface and an SPR angle shift. (**D**) Biolayer interferometry (BLI). The target protein is first immobilized onto the biosensor tip to form a bio-layer, and the biosensor is then dipped into a ligand-containing solution. When ligands bind to the immobilized protein, the thickness of bio-layer will increase. After white light passes through the bio-layer of the biosensor, the transmitted light and reflected light form light interference, which is visualized as an interference spectrum. The change in the thickness of the bio-layer induces a wavelength shift (∆λ). (**E**) Microscale thermophoresis (MST). In a standard MST experiment explored in a capillary, the target protein is labeled with a fluorescent protein or N-hydroxysuccinimide dye. Initially, the fluorescent molecules are distributed freely and uniformly. Then, a microscopic temperature gradient is induced by an infrared laser that causes the fluorescent proteins to move away from the heated area and reach an equilibrium state. As the thermophoresis progress, the fluorescence decreases. After turning off the infrared laser, the fluorescent molecules return to a uniformly distributed state and the fluorescence returns to its initial level. (**F**) DNA-encoded library (DEL) technology. The target protein is first anchored onto a solid support, usually magnetic beads, and then, the target protein is incubated with the compound library. After thorough washing, the high-affinity ligands that remain bound the target protein and their corresponding structures are analyzed. The DNA barcodes are sequenced, decoded, and resynthesized, and the affinity binding assay is repeated to validate the hits.

Thermal shift assay (TSA, also called fluorescence-based thermal shift assay) is a fluorescence-based biochemical method to measure protein thermal stability and was developed in 2001 for HTS drug discovery [42]. It is commonly used to investigate protein–ligand interactions and ligand screening. Proteins usually exist in a native state and will transform into a denatured state that exposes hydrophobic groups upon heating [43]. The exposed hydrophobic groups can bind to the fluorescent dye SYPRO orange, which is quenched in an aqueous environment and unquenched after binding to a protein [44,45]. Upon temperature-dependent conformational change, the amount of exposed hydrophobic group increases, and the stability of the protein is reflected by the fluorescence intensity of the dye [42]. As shown in Figure 4A, a fluorescence–temperature graph can be drawn, and the T_m_ (defined as the midpoint of the protein unfolding transition) reflects protein stability. When a ligand binds to a protein, the stability of the protein can increase, and result in a higher required temperature to fully expose the hydrophobic groups. Consequently, the fluorescence–temperature curve shifts to the right [46].

The small-scale and high-throughput characteristics of TSA make it an excellent platform for discovering small-molecule ligands. Additionally, it is a preferable choice for PPI inhibitor screening. Compared to other ligand screening technologies, the TSA is simple to operate because of its uncomplicated reagents and the minimal time required. In addition, it can be easily miniaturized to a 384- or 1536-well format and is compatible with automation, resulting in good amenability to high-throughput screening. Notably, this method is not suitable for all proteins, because some lack ideal thermal denaturation curves, and thus, stability information cannot be extracted. It has been estimated that 15–25% of recombinant proteins yield unsatisfactory denaturation curves [47]. The lack of tight spherical folds (e.g., inherent obstacles), exposed hydrophobic cores, hydrophobic protein plaques, and relative stability at room temperature are the main reasons for its limitation.

### 3.2. Isothermal Titration Calorimetry (ITC)

Isothermal titration calorimetry (ITC) is usually used to investigate protein–ligand interactions, including the interaction between PPI inhibitors and their target protein [48,49,50,51]. The formation of protein–ligand interactions is a reversible thermally-driven process that involves the absorption or release of heat, which can be detected by calorimetric measurements. The heat change in a reaction at a constant temperature can be quantified by ITC, following the principles of power compensation. Typically, ITC instruments are composed of a sample cell and a reference cell, both of which are coated by an adiabatic jacket [52]. In the sample cells, one component of the PPI is diluted into a solution that contains the other component, and this leads to the absorption or release of heat. The temperature difference between the two cells is detected by a connected sensitive thermocoupled circuit [53]. In detail, as shown in Figure 4B, the vertical axis of an ITC graph shows change in heat per unit time (in cal/s), and the abscissa axis represents the time. As the titration progresses, the system fits the output to a heat-release curve, and thermodynamic parameters can be calculated, to characterize ligand binding.

ITC is one of the most effective experimental methods for interaction investigations, with many merits, including: (1) High sensitivity and accuracy: ITC is suitable for any reaction, including weak interactions, and is not prone to false positive results. (2) Stability: ITC operates stably at a given temperature. (3) High adaptability: the reaction does not require coupling reagents or protein labeling. (4) Recyclability: the samples are not damaged during the titration. Because of the long time required for each titration and the large amount of protein required (~0.5 mg), ITC is generally used for secondary screening. In addition, the measurement range is limited, such that reliable measurement K_D_ values range from 0.001–100 µM [54].

### 3.3. Surface Plasmon Resonance (SPR)

Surface plasmon resonance (SPR) is an optical-based detection technology that was first used for real-time monitoring of binding interactions between two or more molecules in the 1990s [55,56,57]. As shown in Figure 4C, SPR is a physical optical phenomenon that occurs when polarized light hits a prism coated by a thin metal layer (such as gold film) at a specific angle of incidence [58]. Under resonance conditions, the reflected light attenuates sharply, and the angle corresponding to the minimum reflection is defined as the SPR angle and strongly depends on the refractive index of the material near the metal surface [59,60]. In an SPR assay for ligand–protein interactions, the protein is first immobilized onto a sensor chip by covalent coupling or interaction with specific fusion tag-containing antibodies [61]. Then, the ligand solution flows across the surface of the chip, through the microfluidic channel, and continuously binds to the protein. This results in a change in the refractive index of the metal surface and an SPR angle shift. Finally, biophysical data, such as affinity, kinetics, and thermodynamics, are obtained by fitting the sensorgram data to a suitable binding model [59].

The SPR is a powerful method for studying biomolecular interactions and has a wide range of applications. It can be applied to any kind of molecule, from ions and small molecules (organic compounds) to biomacromolecules (including proteins, nucleic acids, glycoproteins, and even whole cells). This method can dynamically detect the interaction of biomolecules and obtain real-time reaction rates and kinetics [57]. In addition, it is unaffected by the color of analytes (turbid, opaque, or colored solutions can be analyzed), thereby reducing the occurrence of false positives. However, temperature fluctuations and non-specific interaction between analytes and sensor surfaces may interfere with the analysis and should be monitored [62].

### 3.4. Biolayer Interferometry (BLI)

Biolayer interferometry (BLI) is another optical analytical approach for measuring macromolecular interactions that is based on the principle of light interference [63]. As shown in Figure 4D, the target protein is first immobilized onto the biosensor tip to form a bio-layer, and the biosensor is then dipped into a ligand-containing solution. When ligands bind to the immobilized protein, the thickness of the bio-layer will increase [64]. After white light passes through the bio-layer of the biosensor, the transmitted and reflected light result in interference, which is visualized as an interference spectrum. Furthermore, the change in the thickness of the bio-layer induces a wavelength shift (∆λ) [65,66]. By measuring the shift of the interference spectrum in real time, the binding specificity, rates of association (*k*_on_) and dissociation (*k*_off_), equilibrium constants (*K*_D_), and analyte concentration can be monitored with precision and accuracy [67,68,69].

BLI technology has unique properties, including: (1) Real-time measurement of the dynamic data of biomolecule interactions. (2) High-throughput: BLI can simultaneously detect up to 16 samples. (3) High sensitivity: the interference pattern is unaffected by unbound molecules or solutions. (4) Wide range of affinity: BLI can measure affinities ranging from millimolar to picomolar, including weak-affinity interactions and instantaneous interactions. (5) Low cost: compared to SPR technology, it does not require microfluidics. Notably, nonspecific interactions between analytes and biosensors should be avoided. In addition, detergent-containing buffers should be exempted for detergents that may form heterogeneous micelles and change the bio-layer unevenly [66].

### 3.5. Microscale Thermophoresis (MST)

Microscale thermophoresis (MST) was developed by Nano Temper Tech. in 2010 and is popularly used in the study of PPIs [70]. MST detects the movement of molecules along a microscopic temperature gradient, which is highly sensitive to the size, charge, conformation, and hydration shell [70]. As shown in Figure 4E, in a standard MST experiment explored in a capillary, the target protein is labeled with a fluorescent protein (such as green fluorescent protein) or N-hydroxysuccinimide dye [71,72]. Initially, the fluorescent molecules are distributed freely and uniformly. Subsequently, a microscopic temperature gradient is induced by an infrared laser, which causes fluorescent proteins to move away from the heated area and reach equilibrium. As thermophoresis progresses, fluorescence decreases. After turning off the infrared laser, the fluorescent molecules return to a uniform distribution, and the fluorescence returns to the initial level [73,74,75]. Once a ligand binds to the protein, the properties of the protein change, leading to differences in protein thermophoresis. This change in the MST signal can be used to analyze the interactions between molecules and calculate affinity parameters.

MST offers the following advantages for studying PPIs [73,75]. (1) Low sample consumption, in which each sample can be as low as 6 µL. (2) Strong adaptability: the assay solutions can range in properties, including biological liquids, such as serum and cell lysates, and DMSO-containing solutions. (3) Short analysis time: all analyses are completed within 10 min. (4) Wide temperature range: analysis possible from 20 to 45 °C. (5) Real-time detection. (6) Wide concentration range: affinities ranging from picomolar to millimolar can be analyzed. (7) Wide analyte molecular size range, from 100 Da to 1 mDa. (8) Low cost. However, it is difficult to obtain accurate information on stoichiometry from MST data, and hence, it is often assumed that the ligand and labeled receptor interact with a 1:1 stoichiometry by default [76].

### 3.6. DNA-Encoded Library

DNA-encoded library (DEL) technology was developed by Smith in 1985, as a phage-based technology for screening target polypeptides or proteins. Gregory Winter also screened artificial antibodies using this technology [77]. As shown in Figure 4F, a variety of genetic information of antibodies is integrated into the phage genome, and millions of antibody fragments (such as scFv fragments) are expressed on the phage surface following translation, and some of these antibodies may bind to the target protein. Inspired by these characteristics, many high-affinity antibody molecules have been obtained and DNA-encoded compound libraries have emerged. Unlike the above-mentioned gene-encoded proteins, this strategy involves gene-encoded chemistry [78]. Phage-displayed proteins take advantage of the biosynthetic link between nucleic acids and protein, that is, the inserted gene will define the protein sequence. However, this biological link is not required in DNA-encoded chemical libraries that only use DNA tag sequence to define chemistry. Amplified DNA barcodes are arranged in combination with each other to form an unprecedented library of compounds that contain DNA tags, which allow molecules to be amplified and identified from very large libraries and relative quantification. DEL screening is performed in a single tube, by incubating the target protein with a compound library. The target protein can be anchored to a solid support, typically a magnetic bead. After thorough washing, only high-affinity ligands remain bound to the target protein [79].

Compared with conventional HTS libraries, DNA-encoded libraries have the following advantages. (1) They are very easy to maintain and use: a DNA-encoded library can be stored in a single detection tube, whereas HTS libraries require large automated facilities. (2) DELs shorten the screening cycle of hundreds of millions of compounds by two-to-three years. (3) The number of compounds in a DEL library can reach hundreds of millions or more, and traditional compound libraries are typically less than 10 million. (4) Compounds in DEL libraries have much higher spatial coverage than traditional compounds. (5) DEL technology can screen drug targets that are difficult to screen using traditional technologies. However, certain problems have been associated with DEL technology, such as the limited type of reactions that are compatible with DNA chemistry and compound yields. Furthermore, in the process of traditional activity screening, changes in protein activity resulting from compound interaction are tested. Often, binding activity dose not equate to functional inhibition.

## 4. Competition-Based Methods

### 4.1. Fluorescence Polarization (FP)

**Figure 5:**
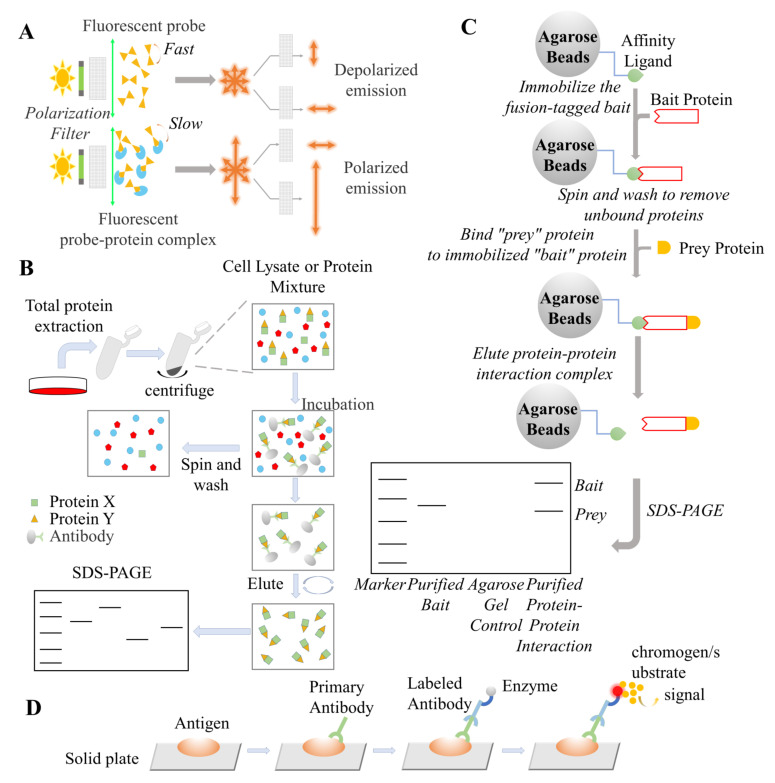
Illustration of competition−based PPI analysis methods. (**A**) Fluorescence polarization (FP). When a fluorophore is excited by polarized light, the emitted light will largely depolarize due to the rapid Brownian molecular rotation of the labeled species during the lifetime of its excited state. Larger molecules emit higher polarized light due to their relatively slower rotation, and smaller molecule emits less polarized light, due to their relatively faster rotation. (**B**) Pull-down. purified bait protein (X) is first immobilized on labelled beads through affinity tags and then, X-bead complexes are incubated with cell lysate. After a series of washing and elution steps to remove the unbound proteins, prey protein (Y) is pulled down. Finally, a Western blotting analysis is carried out to detect the amount of bound prey. (**C**) Co-immunoprecipitation (Co-IP). First, cell lysate is incubated with anti-bait (X) protein antibody-labeled beads. X is immunoprecipitated with the antibody, and the proteins (Y) that binds to X in vivo will also be precipitated at the same time. Thus, both bait (X) and prey (Y) are captured or precipitated on the bead support. After a series of washing and elution steps to release X–Y complexes, the complexes are analyzed by Western blotting with anti-prey antibody. (**D**) Enzyme-linked immunosorbent assay (ELISA). A POI (X) is noncovalently coated onto a polystyrene microwell plate through physical adsorption. The cell lysate is then incubated in the wells and allowed to interact with protein X. Then, a wash step is conducted to remove unbounded protein, and detection antibody is added against the bound protein (Y). The detection antibody is conjugated with an enzyme (enzyme-linked antibody) that catalyzes its substrate to produce a chromogenic or fluorescent product. Finally, the substrate is added to the reaction, and the amount of binding partner bound to X is quantified by an absorbance- or fluorescence-based readout.

The fluorescence polarization (FP) method is usually used to detect interactions between a fluorescent probe-labeled molecule and a protein of interest, and was originally proposed by Francis Perrin in 1926 [80,81,82,83]. As shown in Figure 5A, when a fluorophore is excited by polarized light, the emitted light will be largely depolarized during the rapid Brownian molecular rotation of the labeled species that occurs within the lifetime of the excited state [84]. The polarization degree of the emission light is inversely proportional to the rotation of the labeled molecules. That is, a larger molecule emits more polarized light because of its slow rotation, and smaller molecules emit less polarized light, due to their relatively faster rotation. The FP assay was developed as a technology for PPI inhibitor screening based on competitive binding between fluorescent probes and inhibitors with larger target proteins. Commonly used fluorophores include fluorescein isothiocyanate (FITC), Texas Red, Alexa 488, cyanine-5 (Cy5), rhodamines, tetramethylrhodamine (TAMRA), fluorescein amidite (FAM), and boron-dipyrromethene (BODIPY) dyes [85,86,87]. When the binding affinity of the inhibitor is larger than that of the fluorescent probe, more fluorescent probes are free in solution and less polarized light is detected by a microplate reader equipped with polarizing optics. Using a mathematical equation, the binding affinity constants (*K*_i_ values) of the inhibitors can be calculated [88]. The principle of IMAP using an FP readout involves the addition of binding solution after the kinase reaction with a fluorescently labeled peptide. The small phosphorylated fluorescent substrate binds to large M(III)-based nanoparticles, which reduces the rotational speed of the substrate and thus increases its polarization. IMAP technology provides a homogeneous assay that is applicable to a wide variety of kinases, phosphatases, and phosphodiesterases, as it is not specific for substrate peptide sequences. Furthermore, as IMAP assays are not antibody-based, they are generic and can be used for any kinase, phosphatase, or phosphodiesterase. IMAP assays produce robust fluorescence signals and provide reliable results with good Z-factors.

FP is an economically fast method. In addition, it has many other advantages, including the following (Table 3): (1) Simple operation without a washing step to separate the unbound molecules. (2) HTS is easily conducted in a microwell plate (96-, 384-, or 1536-well). (3) Real-time homogeneity: FP is carried out in solution and is truly homogeneous. Thus, FP can effectively simulate an equilibrium environment and allow real-time kinetic detection. (4) Friendly to the experimenters: compared to radioisotope research methods, this technology is safer and more reliable. Inevitably, it also has disadvantages, including interference from light scattering, quenching, and auto-fluorescence, which can be effectively reduced by selecting longer-wavelength (red-shifted) fluorophores [87,89,90].

### 4.2. Pull-Down

Pull-down assays are widely used to study interactions between a fusion tag-labeled protein of interest (POI, bait) and its potential interacting partners (prey) in vitro and was originally devised by Kaelin et al. in 1991 [91]. Commonly used fusion tags include glutathione S-transferase (GST), polyhistidine, HaloTag (a modified dehalogenase), and biotin [92,93,94,95].As shown in Figure 5B, the purified bait is first immobilized on label-coupled beads through an affinity tag [92]. Then, bait–bead complexes are incubated with prey-containing mixtures (such as a cell lysates). After a series of washing and elution steps to eliminate unbound prey, the prey proteins are pulled down. Finally, Western blot analysis was performed to detect the amount of bound prey. In PPI inhibitor identification, potential PPI inhibitors are added to the prey-containing mixture to compete with prey for binding to bait proteins. Hence, the level of bound prey decreases, which reflects the degree of interruption of the bait–prey interaction.

Pull-down assays, as well as PPI inhibitor screening, have been applied for both PPI identification and confirmation. Pull-down assays require a larger quantity of bait protein than is typically available under endogenous expression conditions to eliminate confounding results that arise from the interaction of the bait with other interacting proteins present in the endogenous system that are not under study. However, the pull-down experiment is a biochemical reaction carried out in a test tube, which does not fully reflect the true state of intracellular protein interaction. Furthermore, the fusion-expressed GST tag has a longer peptide chain, which may change the native fold of the target protein structure (Table 3).

### 4.3. Co-Immunoprecipitation (Co-IP)

Co-immunoprecipitation (co-IP) is a robust and effective method for determining the endogenous interaction between two proteins in intact cells [96,97,98,99]. Similar to pull-down assays, co-IP also involves immobilizing bait proteins on agarose or magnetic beads. The difference in co-IP is that the interaction between baits and beads is based on the specific antigen–antibody reaction. In the basic co-IP experiment, cells are lysed under non-denaturing conditions to maximize the retention of protein–protein interactions in intact cells [100]. As shown in Figure 5C, the cell lysate is then incubated with anti-bait (X protein) antibody-labeled beads. X is immunoprecipitated with the antibody against protein X, and protein Y, which binds to X in vivo, is precipitated simultaneously. Therefore, both bait (X) and prey (Y) are captured or precipitated on the bead support. After a series of washing and elution steps, the X–Y complex is released and analyzed by Western blotting using an anti-prey antibody. Hence, the interaction between X and Y in vivo can be determined.

This technology can reflect the real interactions of target PPIs by retaining PPIs in intact cells. However, low-affinity and transient PPIs may not be detected. In addition, the prey proteins may be complexed with chaperones, signaling molecules, structural proteins, cofactor complexes, etc. Thus, the combination of these two proteins may not be direct, and a third partner may form a bridge between them (Table 3).

### 4.4. Enzyme Linked Immunosorbent Assay (ELISA)

Enzyme-linked immunosorbent assay (ELISA) is an immunological technique developed using immunoenzymatic methods and is widely applied in the detection of antibodies, antigens, proteins, glycoproteins, and PPIs. To study the interaction between proteins, a POI (X) is noncovalently coated onto a polystyrene microwell plate through physical adsorption and maintains its ability to bind to partner proteins. As shown in Figure 5D, the binding partner (Y)-containing solution is then incubated in the wells and allowed to interact with X. Then, a washing step is conducted to remove unbound Y protein, and a detection antibody is added against the binding partner. The detection antibody is conjugated to an enzyme (enzyme-linked antibody) that catalyzes its substrate to produce a chromogenic or fluorescent product. Commonly used enzymes include horseradish peroxidase (HRP) or alkaline phosphatase (AP). Finally, the substrate is added to reaction, and the amount of the binding partner bound to X is quantified by absorbance- or fluorescence-based readout [101]. In PPI inhibitor identification, the potential inhibitor will compete with Y for binding to X, and the IC_50_ value can be calculated according to the readout signal by titrating the inhibitor concentration. ELISA can also adopt an indirect or sandwich format that involves more antibodies.

ELISA is a highly sensitive technology that amplifies signals through enzyme-catalyzed reactions to produce enhanced color or fluorescence [102]. However, it is time-consuming, requires multiple incubation and washing steps, and weak interactions are difficult to detect (Table 3) [30].

## 5. Structure-Guided Methods

### 5.1. Tethering

**Figure 6:**
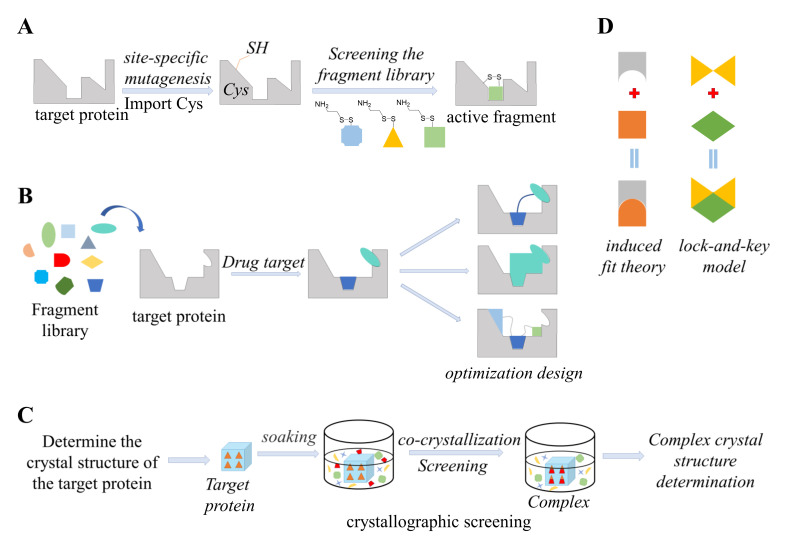
Illustration of structure−guided analysis methods. (**A**) Principles of tethering. First, experimentalists investigate whether there are endogenous cysteine residues near the active pocket of the target protein. If not, cysteine residues are introduced by site-directed mutagenesis. Then, the fragments in the fragment library are all attached to the same sulfhydryl side chain, and the target protein is placed in a high-concentration solution of the fragments. In the solution, the formation and dissociation of disulfide bonds between the fragments and target proteins reach a dynamic equilibrium and active pockets form stable fragment–target protein covalent complexes. (**B**) Principles of fragment-based drug design (FBDD). A fragment library is screened by sensitive detection technology, and the fragments that bind to the drug target are identified (the binding force is weak, generally at the mM level). Then, the structural information of the fragment binding to the drug target is determined. Next, researchers investigate the binding region of the fragment on the drug target and the interaction mechanism. According to structural information on the interaction between the fragment and the drug target, investigators can guide the optimization and derivatization of the fragment and connect the fragments that localize to different binding sites to construct new molecules. (**C**) Principles of crystal soaking. The ligand-free target protein is soaked with the ligand-containing stock solution to obtain the crystal structure of the complex. (**D**) Principles of docking. The ligand molecule is placed at the active site of the receptor, and then, computational methods are used to evaluate the strength and weaknesses of the interaction between the ligand and the receptor in real time, according to the principles of geometric complementarity and energy complementarity. The best binding pose between the two molecules is determined.

Tether technology was invented by Sunesis Company and uses mass spectrometry to identify fragments to guide drug design. Tether technology can not only detect whether a fragment binds to the target protein, but can also detect whether it binds to a specific site. As shown in Figure 6A, fragments containing disulfide bonds screened by the tether method are composed of three parts: the fragment parent, linking group, and leaving group (usually 2-triethylamine). In addition, the active fragments that connect adjacent sites are detected and identified using secondary tether technology, and the disulfide bonds that connect the two fragments are replaced with appropriate linkers to obtain highly active compounds.

The advantages of the tether method are as follows: (1) A stable and reversible disulfide bond is formed between the fragment and the drug target. Through the principles of thermodynamic equilibrium, fragments that are in good agreement with the drug target can be quickly detected by mass spectrometry, which reduces the false-positive rate. (2) The formation of a disulfide bond between the fragment and the drug target is beneficial for carrying out molecular simulations or determining the crystal structure of the complex, to accurately locate the position of the fragment in the active site of the drug target and guide the optimization or connection of the fragment. However, the disadvantage is that it is necessary to introduce cysteine residues into the active site of the drug target by site-directed mutagenesis, which increases the difficulty of the experiment.

### 5.2. Fragment-Based Drug Design (FBDD)

Fragment-based drug design (FBDD) is a new method of drug discovery that combines fragment screening with structure-based drug design. As shown in Figure 6B, FBDD methods first screen low-molecular-weight and low-affinity fragments and then optimize or connect the fragments based on the structural information of the drug target, to obtain new molecules with high affinity for the drug target and strong drug-like properties [103]. In general, fragment-based molecular design can be divided into three stages. (1) Fragment screening: the construction of a fragment library needs to consider three factors, library capacity, chemical structure diversity, and drug-likeness. (2) Obtaining structural information: structural information of the fragment binding to the drug target plays a crucial role in guiding the conversion of the fragment into a lead compound. At present, the technologies mainly used to identify fragment compounds include: surface plasmon resonance (SPR), nuclear magnetic resonance (NMR), X-ray single-crystal diffraction (X-ray), and mass spectrometry (MS). (3) Building new molecules based on fragments. The ultimate goal of fragment-based molecular design is to discover highly active lead compounds and drug candidates.

Fragment-based drug design is a new drug discovery method that has gradually been developed to overcome the shortcomings of traditional HTS methods [104]. Notably, HTS methods are often associated with a low hit rate, which makes it difficult to obtain ideal compounds for some drug targets. Furthermore, the drug-like properties of hit compounds can be relatively poor. The individual fragments of active compounds obtained by HTS often cannot bind well to the active pocket of the target protein, and optimization of a single fragment often affects the entire molecule and even changes the binding position of the target. The greatest problem with FBDD is that the activity of the starting compound is very low. Larger molecules are often required to achieve functional activity; however, if the molecule is too large, its absorption and dissolution properties can hinder quantitative measurements. Therefore, targets suitable for FBDD usually require ligands that exceed the molecular sizes of traditional drugs. Not as many approved drugs have been discovered by FBDD as those that have been identified through HTS methods, and FBDD is not applicable to all HTS targets.

### 5.3. Crystal Soaking

Through co-crystallization and soaking techniques, target proteins can rapidly recognize and bind to active fragments to form complexes, whose three-dimensional structures can be rapidly determined by high-throughput X-ray diffraction techniques. This is known as crystallographic screening. As shown in Figure 6C, Crystallization screening is generally divided into the following stages. First, the crystal structure of the target protein is determined, and a crystallization screening technology platform is established. Then, the protein crystals are “soaked” in the screening fragment mixture to form a fragment–target protein complex for structural testing. The fragment library used for crystallization screening generally contains approximately 1000 molecules, and usually, 10–50 active fragments can be found, from which 4–5 fragments can be selected for further optimization [105].

Crystallization screening is an ideal fragment-based drug-design method. This method can not only detect whether the fragments are bound, but also accurately detect the specific position of the target protein and the three-dimensional fragment-target–protein complex. Structural information not only greatly reduces the probability of non-specific binding and false positives, but also provides direct guidance for subsequent fragment optimization and ligation. The disadvantage of crystallization screening is that it requires advanced technology and instruments, not only to establish a high-throughput protein crystal structure testing platform, but to enable a high degree of experimental automation.

### 5.4. Computational Docking

Computational molecular docking technology is supported by stoichiometry and other disciplines to simulate the geometric structure of molecules and intermolecular forces for identifying intermolecular interactions and predicting the structure of receptor–ligand complexes. The basis of molecular docking is the structure of biological macromolecules, and this structural information can usually be obtained by X-ray crystallography NMR, and homology modeling methods [106]. As shown in Figure 6D, An early principle used to elucidate the ligand–receptor binding mechanism was the “lock-and-key model” proposed by Fisher in 1894. The manner in which a ligand enters a receptor is similar to a lock and key, and the receptor and ligand are considered rigid structures. Namely, in the process of molecular docking between the ligand and receptor, the spatial conformation does not change. However, the actual process of identifying drug and target protein molecules is much more complicated than the lock and key model. First, during the molecular docking process, the receptor and ligand are flexible; that is, the molecular conformation of the target enzyme and inhibitor changes during the binding process. Second, in molecular docking, the receptor and ligand must not only match in spatial shape but also in energy. Notably, the change in binding free energy determines whether they ultimately match. Finally, in the process of mutual recognition between receptors and ligands, there is also a series of interactions between the two, such as hydrogen bonding, electrostatic interactions, hydrophobic interactions, and van der Waals interactions. Due to the limitations of the “lock–key” principle, DE Koshland put forward the “induced fit” theory in 1958, the core content of which is that the binding site of the protein changes in conformation through interactions with the ligand, that is, the protein and the substrate fit snugly together. Structural changes occur, and this principle explains why during molecular docking, both the ligand and the receptor are treated as flexible structures. The docking results obtained by treating ligands and receptors as flexible structures are relatively more accurate [107].

In recent years, molecular docking technology has been widely used in early virtual screening for drug discovery, drug molecular design, lead compound optimization, drug potential target discovery, drug–target interaction mechanisms, and searches for important drug-metabolizing enzymes. However, there remain many problems to be solved. (1) The true three-dimensional spatial structure may be unknown. When the mechanism of action of the receptor is studied on a computer, its spatial conformation changed because it is separated from its native environment. In addition, some receptors have been identified through pharmacological experiments, and their true structures are not yet known. (2) When the molecules are docked, the binding site of the target protein is not necessarily correct, and the screened small-molecule database is not necessarily appropriate. (3) The computational data may not match the experimental data on the real state of the receptor. In the molecular docking experiment, we simulated the experiment on a computer and obtained very detailed data, but it may not be possible to successfully perform molecular dynamics simulations with high scores.

## 6. Functional-Based Methods

### Protein Fragment Complementation Assays (PCAs)

**Figure 7:**
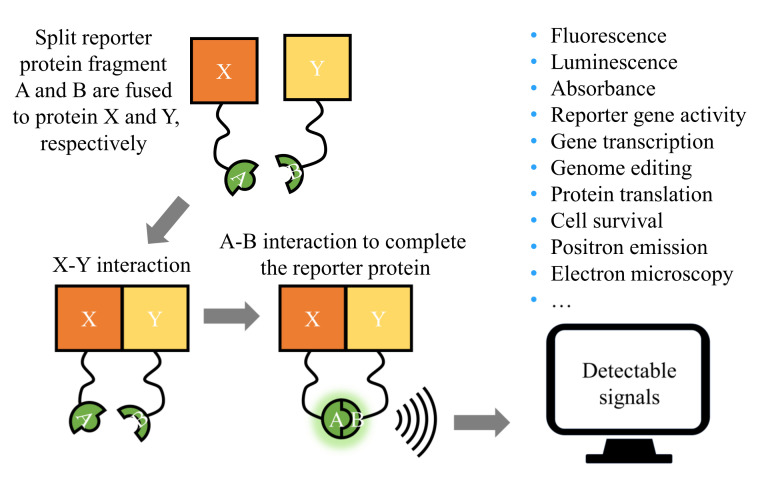
Illustration of protein fragment complementation assays (PCAs). The engineered split reporter protein fragments A and B are fused to proteins X and Y, respectively. Upon interaction of proteins X and Y, fragments A and B noncovalently bind to form a complete reporter protein that can produce detectable signals, such as fluorescence.

Protein fragment complementation assays (PCAs) have been designed to investigate PPIs in cells. As shown in Figure 7, PCA is a method that splits reporter proteins into two interacting fragments that are fused to two POIs. If the two POIs interact, the two reporter fragments combine to produce a detectable signal. Among the reporter proteins, luciferase, and yellow fluorescent protein (YFP), ubiquitin, dihydrofolate reductase (DHFR), β-lactamase, β-galactosidase, TEV protease, GFP, and GFP variants have been engineered. Depending on the specific reporter protein, the detected signals can be based on either fluorescence, luminescence, absorbance, reporter gene activity, gene transcription, genome editing, protein translation, cell survival, positron emission, or electron microscopy.

PCA technology can directly detect molecular interactions both in vivo and in vitro. The results reflect the native protein interactions in the relevant cellular context. In addition, PCA is compatible with automatic high-throughput screening and can be used in any cell line and multicellular organism for PPI studies.

## 7. Case Study

### 7.1. Identification of Type I PPI Inhibitors

**Figure 8:**
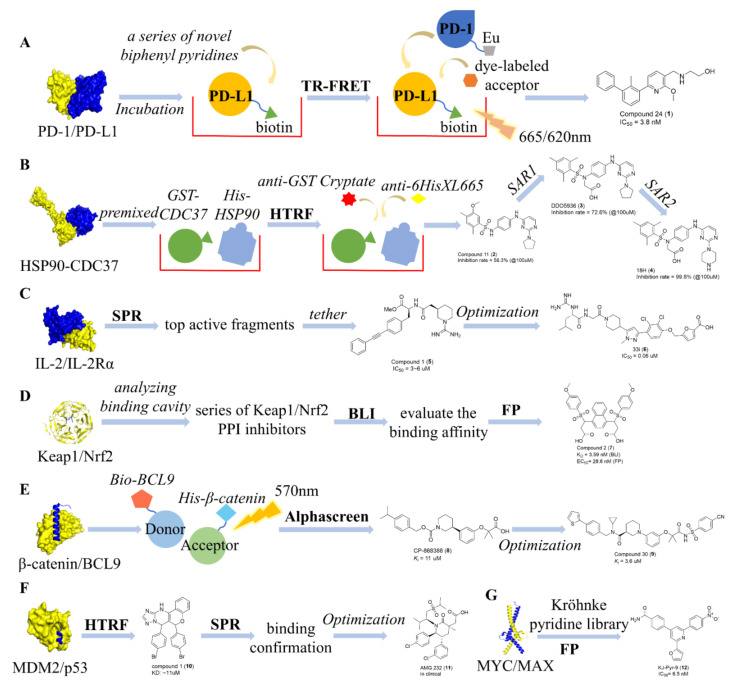
Representative inhibitors of different types of PPIs discovered by current methods. (**A**) PD-1/PD-L1 (type I) inhibitor compound 24 was identified by HTRF technology. (**B**) HSP90-CDC37 (type I) inhibitor 18H was also identified by HTRF technology. (**C**) IL-2/IL-2Rα (type II) inhibitor 33i was discovered by SPR and tether methods. (**D**) Keap1/Nrf2 (type III) inhibitor compound 2 was discovered by BLI and FP strategies. (**E**) β-catenin/BCL9 (type IV) inhibitor compound 30 was identified by AlphaScreen. (**F**) MDM2/p53 (type IV) inhibitor AMG232 was found by the HTRF and SPR methods. (**G**) MYC/MAX (type V) inhibitor KJ-Pyr-9 was discovered by an FP strategy.

Programmed cell death protein 1 (PD-1) is a receptor that is expressed and located on the surface of T cells and B cells. Under normal physiological conditions, PD-1 and its ligand PD-L1 form a complex that inhibits the excessive activation of immune cells and prevents the occurrence of autoimmune diseases. The PD-1/PD-L1 complex is type I PPI. PD-L1 and PD-1 are highly expressed in tumor cells, and their PPI inhibits the activation of the immune system, which helps tumor cells escape the immune system. Therefore, the PD-L1/PD-1 PPI is a hot target for cancer therapy, as blocking PPI can reactivate the immune system and increase its lethality against tumor cells. Although many antibodies targeting the PD-1/PD-L1 PPI have been approved by the FDA, the oncology field is looking forward to the discovery of small-molecule inhibitors that overcome the inherent drawbacks of antibodies, such as long half-life, poor oral bioavailability, and unchecked immune responses. Currently, substantial exploration of small-molecule PD-1/PD-L1 PPI inhibitors is ongoing. In recent years, Wang et al. reported a small molecule, compound 24 (Figure 8A), [108] which was based on a previously reported co-crystal structure of the PD-L1/small molecular inhibitor complex (PDB code: 5J89, 5N2F). The investigators designed a series of novel biphenyl pyridines, and a TR-FRET assay was established to evaluate the PD-1/PD-L1 PPI inhibitory activity of these compounds. In the PD-1/PD-L1 TR-FRET assay, PD-L1-biotin and prediluted compound solution were added and incubated. Subsequently, PD1-Eu and dye-labeled acceptors were added and the resulting solution was incubated. The Tb donor emission was measured at 620 nm, followed by the dye acceptor emission at 665 nm. Data were analyzed using the TR-FRET ratio (665 nm emission/620 nm emission). Ultimately, compound 24 (Figure 8A) with an IC_50_ value of 3.8 nM was discovered by TR-FRET and showed significant in vivo antitumor activity in a subsequent activity evaluation.

Heat shock protein 90 (HSP90) is a chaperone that maintains cellular protein homeostasis. HSP90 and its cochaperones (such as CDC37, HSP70, and AHA1) help mature nascent or misfolded peptides. CDC37 is a kinase-specific chaperone that recognizes and delivers kinases to HSP90, and the HSP90/CDC37 complex forms a type I PPI. CDC37 is overexpressed in tumor cells, compared to normal cells, and CDC37/HSP90 synergistically facilitates the maturation of oncogenesis. Hence, targeting CDC37/HSP90 PPI is a robust strategy for developing a novel cancer therapy [109]. In 2019, our team identified a specific small-molecule CDC37/HSP90 PPI inhibitor, DDO-5936, with antiproliferative activity against colorectal cancer cells [110]. In the screening of hit compounds, an HTRF assay was developed to evaluate the ability of compounds to disrupt these PPIs. In the HTRF assay, GST-tagged CDC37 and His-tagged Hsp90 were premixed in a buffer. The mixture was then incubated with a pre-diluted solution containing compounds. Subsequently, anti-GST cryptate and anti-His_6_-XL665 were added and the cell and the samples were incubated. The donor emission was measured at 620 nm, followed by the acceptor emission at 665 nm. Data were analyzed using the TR-FRET ratio (665 nm emission/620 nm emission). Finally, compound **11** (**3**), was identified, and DDO-5936 (**4**) and 18H (**5**) were obtained for further structural optimization (Figure 8B) [111].

### 7.2. Identification of Type II PPI Inhibitors

Interleukin-2 (IL-2) is a proinflammatory cytokine that plays an important role in the growth of active T cells, through mediating the T-helper 1 (Th1) immune response [112,113,114,115,116]. The interaction of IL-2 and its receptor (IL-2R, a heterotrimeric complex consisting of IL-2Rα, IL-2Rβ, and IL-2Rɣ) induces the proliferation and clonal expansion of activated T cells [117]. Antibodies that target the α receptor subunit (IL-2Rα) to block IL-2 binding have been proven to be clinically effective as immunosuppressive agents [118,119]. Hence, developing small-molecule IL-2/IL-2Rα PPI inhibitors is a potential immunosuppressive therapy strategy. The binding mode of the IL-2/IL-2Rα complex is typical type II interaction, in which protein–protein interactions are driven by a small set of “hot spots.” [120]. Raimundo et al. reported an IL-2/IL-2Rα PPI inhibitor that was developed through fragment-assembly approaches [117,121]. In the process of fragment identification, an SPR assay was used to evaluate the affinity of fragments for IL-2. The top active fragments were then tethered to obtain the hit compound 1 (**5**, IC_50_ = 3–6 μM). Finally, **33i** (**6**, IC_50_= 0.06 μM), with favorable in vitro and in vivo pharmacokinetic properties, was obtained after structural optimizations (Figure 8C).

### 7.3. Identification of Type III PPI Inhibitors

The Nrf2 transcription factor is a member of the CNC protein family. Nrf2 is ubiquitously expressed in tissues and is a major mediator of cellular adaptation to oxidative stress. Kelch-like ECH-associated protein 1 (Keap1) is a component of the ubiquitin ligase complex that is specially targeted for inhibition by both chemopreventive agents and oxidative stress. Nrf2 forms a cytoplasmic complex with Keap1. This complex prevents Nrf2 activation and nuclear translocation by promoting its ubiquitination and proteolysis [122,123,124]. Disruption of the Keap1/Nrf2 PPI has been identified as a promising approach for oxidative and inflammatory stress-mediated cancer and other chronic diseases [122,123]. Keap1/Nrf2 is a typical type III PPI, in which the Nrf2 high-affinity ETGE peptide enters the hydrophobic inner cavity of Keap1 [124]. Jiang et al. discovered a potent Keap1/Nrf2 PPI inhibitor based on molecular binding analysis [66]. After analyzing the Nrf2 binding cavity in Keap1 and binding mode of reported small molecule inhibitors, the investigators designed and synthesized a series of Keap1/Nrf2 PPI inhibitors. A BLI assay was used to evaluate the binding affinity of the compound to Keap1, and an FP assay was used to further validate binding. Finally, compound 2 (**7**, *K*_D_ = 3.59 nM (BLI), EC_50_ = 28.6 nM (FP)) was identified as the most potent inhibitor in subsequent functional experiments (Figure 8D).

### 7.4. Identification of Type IV PPI Inhibitors

In 2014, Zhang et al. discovered a β-catenin/BCL9 PPI inhibitor, CP-868388, using the AlphaScreen method [125]. Oxidative stress can activate the transcription factor Foxo, and the interaction between β-catenin and Foxo leads to cell cycle arrest, resting state or apoptosis. Wnt/β-catenin signaling can also affect H_2_O_2_-mediated cell death. The Wnt/β-catenin signaling pathway (also known as the canonical Wnt signaling pathway) participates in multiple physiological processes, including cell proliferation and differentiation, stem cell self-renewal, and tissue homeostasis [126,127,128]. Dysregulation of the Wnt/β-catenin signaling pathway is closely related to tumorigenesis, such as in colon cancer [129,130]. β-Catenin is a pivotal mediator that regulates the Wnt pathway by interacting with other proteins. Aberrant protein–protein interaction between β-catenin and B-cell lymphoma 9 (BCL9) is responsible for the transcriptional overactivation of canonical Wnt signaling, and thus, this PPI is a promising target for cancer therapy. The β-catenin/BCL9 complex belongs to the type IV PPI family, and the α-helical region of BCL9 binds to the first armadillo repeat of β-catenin. Zhang et al. reported an HTS campaign for β-catenin/BCL9 PPI inhibitors using the AlphaScreen method [125]. As shown in Figure X, BCL9 was biotinylated and β-catenin was tagged with His_6_, to attach the proteins to streptavidin-coated donor beads and nickel chelate acceptor beads, respectively. When the donor and acceptor beads were brought within 200 nm, emission light at 570 nm was detected. If a β-catenin/BCL9 PPI inhibitor completely disrupted the PPI, no fluorescence signal would be observed. Finally, the hit compound CP-868388 (Figure 8E) was discovered with a *K*_i_ value of 11 µM. Further chemical structure optimization through scaffold hopping and SAR evaluations was conducted to obtain improved compound 30 (**9**), which selectively inhibits the β-catenin/BCL9 PPI with a *K*_i_ of 3.6 μM in AlphaScreen competitive inhibition assays [131].

The Wnt/β-catenin signaling pathway induces DNA damage response by regulating multiple proteins such as p53. The transcription factor p53, also known as a tumor suppressor, plays a crucial role in cell growth regulation, progression through the cell cycle, apoptosis, and genomic stability. In 50% of human cancers, p53 is defective due to functional mutations or inhibition by its negative regulator, murine double minute 2 (MDM2). Hence, development of an MDM2/p53 PPI inhibitor is a promising strategy for cancer therapy. The MDM2/p53 complex, a type IV PPI, and the α-helical region of MDM2 binds to the N-terminal transactivation domain of p53. In 2009, Allen et al. carried out an HTRF-based high-throughput screen to discover an MDM2/p53 PPI inhibitor. A hit, compound 1 (**10**), with an HTRF IC_50_ of 3.88 μM, was discovered [132]. Subsequently, an SPR assay revealed that compound 1 reversibly binds to MDM2 (K_D_: ~11 μM), and the cocrystal structure of compound 1 bound to MDM2 was obtained. Based on this hit, structure-based rational design and synthesis were performed to obtain potent MDM2/p53 PPI inhibitors with good pharmacokinetic properties and in vivo efficacy [133,134,135]. The most potent inhibitor, AMG 232 (**11**), has entered into clinical trials and is in phase I for the treatment of cancer [135,136].

### 7.5. Identification of Type V PPI Inhibitors

The oncogene MYC (also known as c-MYC) is overexpressed in almost all cancers and has become an important oncotarget [137,138]. Overactivation of MYC in normal epithelial cells produces a large amount of proteotoxic stress and leads to decreased cell viability. MYC dimerizes with MYC-associated protein X (MAX) before eliciting its oncogenic effects by binding to the promoters of target genes to regulate transcriptional activity [139,140,141]. The MYC–MAX interaction is a typical type V PPI, in which a two-helix bundle of MYC interacts with another two-helix bundle of MAX (two α-helices of both two-helix bundles are separated by a loop) [142]. In 2014, Hart et al. identified an MYC/MAX PPI inhibitor from a Kröhnke pyridine library [143]. They adopted an FP assay to screen potential PPI inhibitors and found that Alexa Fluor 594- labeled E-box containing DNA duplex works as a fluorescence probe. Combined with subsequent evaluations of specificity, MYC-dependent transcription, anti-proliferation, and in vivo activity, KJ-Pyr-9 (**12**, IC_50_ = 6.5 nM) was identified as the most potent PPI inhibitor (Figure 8F).

## 8. Conclusions

Oxidative stress is a kind of negative effect produced by free radicals in the body, and is considered to be an important factor leading to aging and disease. The research on oxidative stress has always been a hotspot of basic research. Oxidative stress can cause changes in a variety of cytokines, and the antioxidant defense mechanism will mobilize a variety of signaling factors to fight oxidative stress, such as Nrf2, HSP90, IL-2, etc. Therefore, these proteins are promising targets for the development of antioxidants. In this review, we first divided proteins into five categories according to their modes of action: type I, two proteins interact through preformed surfaces; type II, proteins with preformed globular structures that adapt upon interaction to form a complex with a novel conformation; type III, a rigid globular protein interacts with a peptide; type IV, a flexible globular protein interacts with a peptide; type V, interaction between two peptide chains. Then, we classified PPI analysis methods according to different mechanisms. We described in detail the principles, advantages, and disadvantages of these methods, as well as the types of PPI applicable to each method. Finally, we listed the classic cases of oxidative stress-related protein inhibitor development, and described their development process and analysis methods. We hope this review provides ideas for the selection of methods for the subsequent development of PPI inhibitors.

## 9. Perspectives

During the past few decades, increasing research efforts toward the development of small molecules that target PPIs have emerged and resulted in multiple successful drug candidates entering the clinic. Unlike traditional enzyme-ligand binding modes, PPIs usually exhibit dynamic features that can be classified into five types, based on the binding modes and epitopes of the interaction interface. Although some common biological methods can be used to screen compounds, it is important to distinguish between various methods when designing specific small molecules to target different types of PPIs. In both academia and industry, an increased demand for PPI targets will require rational methods for the development and optimization of small molecules. There is an urgent need for medicinal chemists to understand the current methods that can be applied to multiple types of PPIs. Recognizing the principles and applications of the different methods will benefit the design of PPI inhibitor discovery campaigns and methods for addressing current challenges. Thus, we provide a review of the current methods that are specifically used in the discovery of small molecules that target oxidative stress-related PPIs, including proximity-based, affinity-based, competition-based, and function-based methods. In the future, we expect that the scientific community will develop even better methods for use in the discovery of small molecule inhibitors of different types of PPIs. Overall, with an increasing number of successful examples, new approaches and concepts for the discovery of small molecules that target oxidative stress-related PPIs will be developed and applied and will provide additional insights into the development of PPI inhibitors.

## Figures and Tables

**Table 1:** Comparison of proximity-based analysis method.

Assay	Alpha Technology		FRET Technology		
	Alpha- Screen	Alpha- LISA	FRET	TR-RET	HTRF
Excitation wavelength (nm)	680		436	320	
Emission wavelength (nm)	520–620	615	528	665	
Distance (nm)	<200		<10		
Advantage	Simple operation Broad energy transfer distance High sensitivity High throughput Wide applicability		Simple operation Stability Low interferences Wide range of applications High throughput Homogeneity		
Disadvantage	Large signal fluctuations affected by stray light and temperature “Hook effect”		Relatively limited application Higher requirements for experiments		
Applicable PPI type	I, II		I, II		
HTS suitability	Yes		Yes		

FRET: fluorescence resonance energy transfer; TR-FRET: time-resolved fluorescence energy transfer; HTRF: homogeneous time-resolved fluorescence; PPI: protein–protein interaction; HTS: high throughput screening.

**Table 2:** Comparison of affinity-based methods.

Assay	Advantages	Disadvantages	Applicable PPI Type	HTS Suitability
Thermal Shift	Small scale Fast Automation High throughput	Not suitable for some proteins that have no ideal thermal denaturation curve and stability information cannot be extracted	All types	No
ITC	Sensitive and accurate Stable High adaptability Recyclability	Not suitable for HTS	All types	No
SPR	Dynamic Monitoring Unaffected by the color of analytes High adaptability	Sensitive to temperature Non-specific adsorption	All types	No
BLI	High throughput Real-time measurement High sensitivity Low cost	Non-specific adsorption High false positive rate	All types	No
MST	Strong adaptable Fast Wide temperature range Wide concentration range Wide molecule size range Real-time detection Low cost Less sample consumption	No accurate information on stoichiometry can be obtained.	All types	No

ITC: isothermal titration calorimetry; SPR: surface plasmon resonance; BLI: biolayer interferometry; MST: microscale thermophoresis; HTS: high throughput screening.

**Table 3:** Comparison of competitive-based analysis method.

Assay	Advantage	Disadvantage	Applicable PPI Type	HTS Suitability
FP	Simple operation High throughput Real-time and homogeneity Friendly to experimenters	Interference from light scattering, quenching, and auto-fluorescence.	All types	Yes
Pull Down	Direct protein–protein interactions can be verified Conjugated beads with strong affinity and high elution purity	Cannot fully reflect the true state of intracellular protein interaction, and the fusion expressed GST tag may change the original folding of the target protein structure.	All types	No
Co-IP	Reflect the real interaction of target PPI in intact cells	Low affinity and transient protein–protein interactions may not be detected. May not reflect direct interaction, a third party may act as a bridge in between.	All types	No
ELISA	Highly sensitivity	Time-consuming Weak interactions are hard to detect	All types	No

FP: fluorescence polarization; Co-IP: co-immunoprecipitation; ELISA: enzyme-linked immunosorbent assay; PPI: protein–protein interaction; HTS: high throughput screening.
